# The role of vocal learning in call acquisition of wild grey seal pups

**DOI:** 10.1098/rstb.2020.0251

**Published:** 2021-10-25

**Authors:** Amanda L. Stansbury, Vincent M. Janik

**Affiliations:** ^1^ Sea Mammal Research Unit, Scottish Oceans Institute, University of St Andrews, Fife KY16 8LB, UK; ^2^ El Paso Zoo, El Paso, TX, USA

**Keywords:** vocal learning, grey seals, *Halichoerus grypus*, playbacks, usage learning, production learning

## Abstract

Pinnipeds have been identified as one of the best available models for the study of vocal learning. Experimental evidence for their learning skills is demonstrated with advanced copying skills, particularly in formant structure when copying human speech sounds and melodies. By contrast, almost no data are available on how learning skills are used in their own communication systems. We investigated the impact of playing modified seal sounds in a breeding colony of grey seals (*Halichoerus grypus*) to study how acoustic input influenced vocal development of eight pups. Sequences of two or three seal pup calls were edited so that the average peak frequency between calls in a sequence changed up or down. We found that seals copied the specific stimuli played to them and that copies became more accurate over time. The differential response of different groups showed that vocal production learning was used to achieve conformity, suggesting that geographical variation in seal calls can be caused by horizontal cultural transmission. While learning of pup calls appears to have few benefits, we suggest that it also affects the development of the adult repertoire, which may facilitate social interactions such as mate choice.

This article is part of the theme issue ‘Vocal learning in animals and humans’.

## 1. Introduction

Vocal production learning is widely recognized as a key skill in the development of flexible communication systems [1]. At its most complex, it allows humans to learn novel signals for referential use in language and creates tremendous diversity of song patterns in humans and animals alike. We know that vocal learning is used in song acquisition in songbirds [2], humpback whales [3] and greater sac-winged bats [4]. Delphinids and a variety of birds and bats use learned signals in individual, group and mother–offspring recognition [5]. Vocal production learning also influences context-specific calls, such as rain and alarm calls in birds [6]. The extent to which vocal learning is used in some mammals is an open question and for some vocal learners such as elephants [7] and pinnipeds [8] very little information is available.

Vocal learning in mammals has received considerably less research attention than it has in birds. While birds have many convergent adaptations that allow us to use them as a model system for the study of vocal learning in general, the brain structures used are not homologous to those of humans [9]. Among mammals, only cetaceans, pinnipeds, bats, elephants and humans have clear vocal production learning capabilities [10]. To date, we know very little about call development in mammalian vocal learners and whether their entire call repertoire is influenced by learning or not. This knowledge is crucial to assess similarities between humans and other mammalian vocal learners and to work towards the development of a mammalian model for the acquisition of communication signals.

The only mammals that are convincingly capable of modifying formant frequencies, demonstrating vocal learning most similar to a human speech by matching vowel sounds, are elephants [11] and phocid seals [8]. So far, these are the two groups that have received the least research attention among mammalian vocal learners. To address this gap and start the investigation of contextual use as well as the development of learned signals in pinnipeds we addressed the following two questions: Does the acoustic environment of pups influence their call development and how does the context in which a call is heard affect learning? Specifically, we asked whether pups are more likely to copy sounds heard when feeding since these could indicate food availability and may be useful to elicit nursing. Grey seals primarily use a distinctive pup call early in life in the context of mother–pup interactions [12]. Pups are born on land and stay with their mothers for the first 2–3 weeks [13]. After mothers return to sea, pups spend another 10–28 days onshore before starting to forage at sea by themselves [14]. The acoustic inputs that seal pups are exposed to early in life are mostly other pup calls as well as some adult grey seal and sea bird calls.

## 2. Methods

### (a) Subjects

We studied 12 wild grey seal pups (*Halichoerus grypus*, six females and six males) born on the Isle of May (Firth of Forth, Scotland) in November 2011 (pups A, B, C and D), 2012 (pups E, F, G and H), and 2013 (pups I, J, K and L) (table 1). Pups were opportunistically selected as focal animals based on location and birth date. Only animals near the periphery of the colony were chosen to minimize disturbance to the seals. Researchers observed all focal pups being born or just after birth (as indicated by blood on the pup's fur and the presence of the placenta nearby). Recordings from 2011 served as controls and provided baseline information on pup vocalizations without playbacks. For playbacks in 2012 and 2013, we used an additional proximity criterion such that two individuals were selected together. These two pups had to be born within 24 h of each other and within 10 m of each other's pupping site but to different mothers, allowing two pups to receive sound playbacks simultaneously at approximately the same age. To enable different playback stimuli to be used in the same breeding season without interference, two such pairs of seals in each year were chosen from opposite ends of the breeding colony (at least 200 m apart). During each recording session, the identity and relative location of all animals within 30 m of the focal pup were recorded. Adults were identified by unique coat patterns [15] and pups were identified through association with their mother, approximate developmental stage and location.

**Table 1.** Total number of calls analysed per animal. Four pups were recorded each year for 3 years. For each pup, playback condition (type of sound stimuli played to the animal), behavioural condition (context in which sound stimuli were played) and sex are noted.

pup ID	year	sex	playback stimulus	behavioural condition	total no. calls recorded	no. playback sessions
A	2011	M	none	control	349	0
B	2011	M	none	control	404	0
C	2011	F	none	control	701	0
D	2011	F	none	control	56	0
E	2012	F	*a*	feeding	416	15
F	2012	M	*a*	varied	781	12
G	2012	F	*b*	feeding	685	15
H	2012	F	*b*	varied	861	16
I	2013	F	*a*	feeding	206	12
J	2013	M	*a*	varied	49	13
K	2013	M	*ab*	feeding	489	14
L	2013	M	*ab*	varied	177	10

### (b) Acoustic recordings and sound playbacks

Daily in-air acoustic and video recordings were made of each focal pup using a Sennheiser MKH 416 P48 directional microphone (frequency response 40 to 20 kHz, sensitivity at 1 kHz 25 mV Pa^−1^ ± 1 dB) and a Marantz Pro Solid-state recorder PMD671 (sampling rate 96 kHz, 24 bit). Concurrent video recordings were taken using a Sony DCR-HC96E digital video camera. Each pup was recorded for between 30 and 120 min each day from birth, depending on weather. Recordings and playbacks were not conducted in adverse conditions (rain and/or wind over 25 mph). Recordings were limited to daylight hours (approx. 7.00–17.00) to minimize disturbance to the colony and for the researcher's safety. Without visibly disturbing seals, the recording equipment was placed no more than 10 m from the focal animal. A researcher observed all recording sessions from either behind a rock wall or a distance of at least 10 m away from the focal seal.

In 2011, four pups were recorded as controls. In 2012 and 2013, a total of eight pups were recorded and received sound playbacks. Of the eight animals that received playbacks, four were played sounds while nursing in the ‘feeding’ context, and four heard the sounds when their neighbouring pup was nursing regardless of their own behaviour in the ‘varied’ context. Playbacks started at 4 days old and concluded upon weaning between two and three weeks of age [13]. A pup was assumed to be weaned when it had not been seen with its mother for 3 days. During playbacks, the two selected pups of the same age at each playback location heard the playback sounds in different contexts but at the same time. One pup heard the playback while nursing, referred to as the ‘feeding context’. The other pup heard the same sound when the neighbouring pup was nursing, regardless of its own behaviour or distance to its own mother, referred to as the ‘varied context’ as that pup's behaviour was variable. As soon as the feeding pup was observed nursing, the researcher initiated playback (this typically took about one minute from the start of nursing). Upon initiating nursing, pups typically nursed for extended periods (longer than the time to complete playbacks). In a very small number of playbacks (5%), pup feeding was interrupted during playback (for example, by a neighbouring animal coming close to the mother/pup). In this case, playbacks were paused until feeding resumed (this always occurred within 2 min of initial start of playback). The total number of stimulus repetitions stayed constant in these cases.

**Figure 1.**
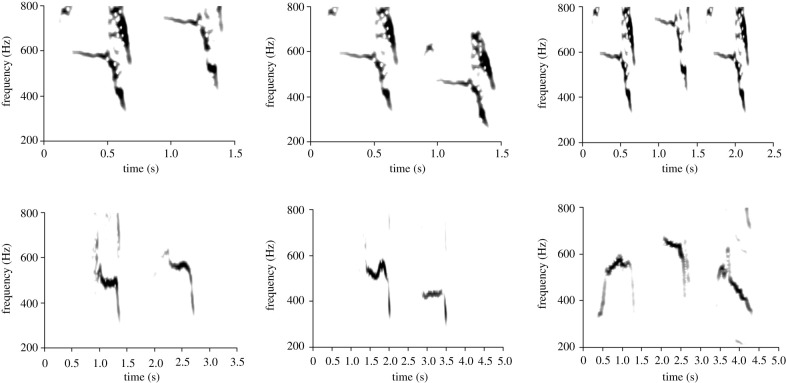
Spectrograms of sound stimuli (upper row) and copies by a pup (lower row). Stimuli were made from a recording of a 4-day-old pup unknown to the playback subjects. Stimulus calls were digitally altered to vary in number (2 or 3) and average peak frequency. Fundamental frequency in stimuli sequences either increased in frequency (left spectrograms, stimulus *a*), decreased in frequency (middle spectrograms, stimulus *b*) or increased and then decreased over three calls (right spectrograms, stimulus *ab*).

Playback stimuli were produced from recorded pup calls on the Isle of May of a 4-day-old pup from a previous breeding season in 2011, which means that none of the study animals had been exposed to it before. Pup calls are distinctive call types in the grey seal repertoire with higher fundamental and peak frequencies and shorter duration than adult calls [16]. Calls were combined into two- or three-call stimulus sequences (figure 1). Calls were digitally altered to vary in average peak frequency using the Adobe Audition 2.0 ‘pitch shifter’ function. This function keeps the duration of the call constant while adjusting the pitch of the call such that the relative frequency structure of the call, including harmonics, remains intact [8]. Pitch was shifted within the limits of a pup's natural repertoire. The initial recorded call had an average peak frequency of 119 Hz. A second call with an average peak frequency of 153 Hz was created from the initial one with the pitch shifter function. Using these two calls we created the stimulus sequences. The first stimulus sequence was a two-call up step in fundamental frequency (stimulus *a*), the second was a two-call down step in fundamental frequency (stimulus *b*), and the third a three-call up then down step in fundamental frequency (stimulus *ab*) (figure 1). Individual calls within each sequence were 0.7 s long, with an inter-call interval of 0.05 s. Four of the playback pups heard stimulus *a*, two heard stimulus *b*, and two heard stimulus *ab* (electronic supplementary material, audio file S1). Stimulus sequences were played in a block of 50 (inter-sequence interval 1 s) in one session per day. In the absence of information on seal sound acquisition, the repetition was chosen based on young songbirds exposed to tutor songs, which learn best when exposed to between 40 and 70 repetitions of a template per day [17]. Playback stimuli were played to animals using a Skytec 170.170 active speaker (frequency response 32–22 000 Hz). The speaker was oriented towards and as close to the focal animal as possible without visibly disturbing the seals (1–10 m from the seal). Playback source levels were adjusted by ear to fall within the normal call source levels for pups, based on comparison between playbacks and call recordings.

### (c) Analysis

Audio recordings were evaluated using Adobe Audition 2.0 (FFT size: 2048, frequency resolution: 46.87 Hz, time resolution: 10.66 ms, weighting function: hamming, window width: 100%). All sounds were high-pass filtered at 100 Hz to remove environmental noise. Each seal's vocalizations were compared with the video feed, and sounds corresponding to visible movements of the face, head, or diaphragm of an identifiable animal were isolated for further analysis. In total, 270 h of audio recordings were analysed, which averaged to 48 h (±5.3) per pup. In total 5174 calls were analysed, averaging 431 (±279) per pup. The number of individual calls recorded for each pup is given in table 1. Individual calls were allowed to have silent breaks of up to 5 ms but calls with larger gaps were counted as two calls. Only tonal, harmonic pup calls were used in this analysis. These calls contained at least one emphasized frequency band but usually also had several harmonics. The number of individual calls was counted and call fundamental as well as peak frequency were measured every 5 ms over a call and then averaged using Avisoft-Saslab Pro 5.02.04 software. Formant frequencies were measured using Praat version 5.3.51. To investigate seal responses, we analysed the vocal behaviour of our target animals for on average 28.9 ± 16.3 min after the end of the playback. Pups were also recorded for on average 44.39 ± 42.21 min before each playback and these data were also included in the analysis since we expected the animals to change their repertoire permanently rather than matching only what they just heard.

To test whether exposure to playbacks increased the chance of producing the provided call-template, a binomial mixed-effects linear regression model was applied, with the dependent variable being whether a call was a match or not. A pup's calling was defined as a match if two or three (depending on stimulus) calls occurred within 1 s of each other and with an average fundamental frequency change of at least 100 Hz between the calls in the same direction as in the call template. This was not an assessment of similarity overall but a measurement of relative frequency change as a variable. Using 100 Hz as a cut-off was successful in a previous study [8]. For control pups, that did not receive any playbacks, any calls produced that matched any stimulus used on playback pups (whether stimulus *a*, *b* or *ab*) was marked as a match. This provided a measure of how often pups produced calls matching stimuli by chance. In the model, individual seal pup was the random effect to control for repeated sampling of multiple calls from each animal. The independent variable was age as a continuous variable interacting with playback condition, either being the control, feeding or variable context.

To test in more detail how closely animals matched the call template and to see if the quality of matches changed with age, the degree of similarity in more specific parameters (electronic supplementary material, table S1) was measured using a dissimilarity matrix with the Mantel statistic. The daisy function and ‘Gower’ distance were used in the *cluster* package for R 1.15.2 [18]. Separate matrices were calculated for the signals played and the seal's response. The Mantel test then measured the association between matrices using Pearson's product–moment correlation coefficient [19]. The Mantel R value was then modelled using a linear mixed-effects model, with variables of individual, gender, age and condition. To measure how values changed by age, age in days was grouped into four categories (0–4, 5–9, 10–14, 15–19 days). In both models, the model parameter estimates were then exponentiated to transform to the scale of the response variable and allow interpretation of odds ratios.

## 3. Results

**Figure 2.**
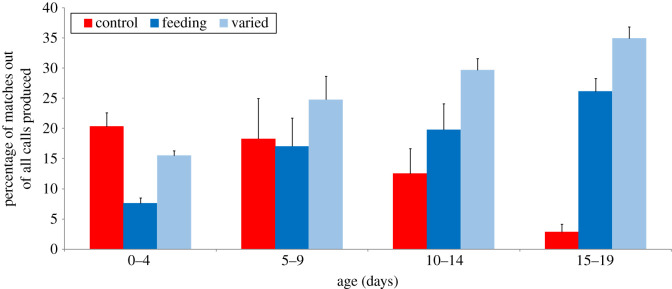
Percentage of pup calls matching the average fundamental frequency of playback stimuli as a function of pup age. For the control pups, which did not receive any playbacks, any calls produced that matched the fundamental frequency change of one of our stimuli (*a*, *b* or *ab*) was counted as a match. This provided an indicator of how often pups produced calls matching stimuli by chance. Pups that received playbacks, either in the feeding or in the varied behavioural condition, were only marked as producing a match if they produced their specific playback stimuli. (Online version in colour.)

Exposure to playbacks increased the chance of pups producing a call matching the playback sound (figure 1; electronic supplementary material, audio file S2). Control pups that did not receive playbacks matched any one of the stimulus frequency changes played to other pups by chance in approximately 20% of the calls they produced when 0 to 4 days old (figure 2). As they aged beyond 4 days, control pups showed fewer matches. By the time of weaning, they produced matches by chance in approximately 2% of calls. This is not an effect of age changes in fundamental frequency since we measured the relative change of fundamental frequency between successive calls here. By contrast, playback pups produced more matches with increase in age and the number of playbacks received. The binomial mixed-effects linear regression model showed a highly significant effect of age, with more matches occurring over time (table 2). From birth to weaning, playback pups produced matches 2 times more often (figure 2). Of the playback pups, those in the varied condition (i.e. heard the playback in varied context when other pup was nursing) produced matches 1.5 times more often than pups in the feeding condition (table 2). At weaning, pups in the varied condition matched their stimulus in approximately 35% of the calls they produced (figure 2).

**Table 2.** Binomial mixed-effects linear regression model examining if exposure to playbacks increased the chance of producing a call matching the playback sound. The dependent variable was if a pup's call was a match, with individual animal as the random effect and age interacting with playback condition as the independent variable.

variable	parameter estimate	CI 2.5%	CI 97.5%	*p*
intercept	1.116	1.088	1.755	< 0.001
age	1.031	1.005	1.198	< 0.005
condition = feed	1.243	0.627	1.464	< 0.08
condition = varied	1.651	1.514	2.781	< 0.01
age: condition = feed	1.544	1.303	1.995	< 0.001
age: condition = varied	1.981	1.576	2.769	< 0.001

**Figure 3.**
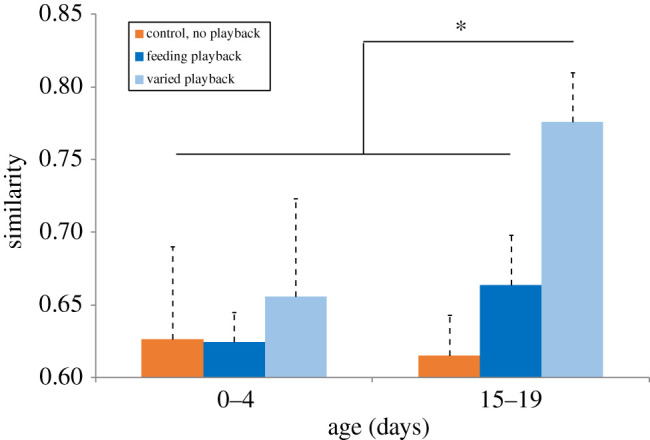
Degree of call similarity, measured using the Mantel statistic, by age for pups in the control condition (receiving no sound playbacks), feeding condition (receiving sound playbacks while nursing) and varied playback (received sound playbacks regardless of behaviour context). Asterisk indicates *p* < 0.01. (Online version in colour.)

The accuracy of matches using multiple call parameters as a measure (electronic supplementary material, table S1) for control pups and pups that heard the playbacks while feeding did not significantly improve with age (figure 3 and table 3). However, calls of pups that heard the playbacks in the varied condition significantly improved with age, with a 25% increase in the degree of similarity to the stimulus from birth to weaning.

**Table 3.** Linear mixed-effects model examining if quality of matches changed with age. Degree of similarity between sound stimuli and seal pup calls was measured using a dissimilarity matrix with the Mantel statistic. The Mantel statistic was modelled with variables of individual, gender, age and condition.

variable	parameter estimate	CI 2.5%	CI 97.5%	*p*
intercept	1.980	1.693	2.472	< 0.001
age	0.998	0.976	1.011	0.438
condition = feed	0.973	0.902	1.015	0.144
condition = variable	0.964	0.912	1.019	0.196
age: condition = feed	1.005	0.993	1.089	0.173
age: condition = variable	1.225	0.738	2.102	< 0.001

## 4. Discussion

Our results showed that grey seal pup call development was clearly influenced by the calls we played, demonstrating a capacity for horizontal cultural transmission. We analysed learning on two levels, an overall measurement of whether the animals' average fundamental frequency changed in the same way as in the template sounds, and more detailed measurement of parameters of the calls analysed in a similarity matrix. Using different stimuli in different parts of the island, we could show that the animals were matching their specific templates over time. The results from the overall average frequency changes only indicate usage learning since control seals, which did not receive playbacks, had the same types of frequency changes in their repertoires. While these frequency changes disappeared over time in control seals, they became more dominant in the experimental seals. However, pups also successfully matched different stimuli more closely, with multiple pitch parameters changing in opposite directions (between stimuli conditions), suggesting that the animals were using their production learning skills to produce copies. Interestingly, pups also changed their overall call structure from single long continuous vocalizations before playbacks to two or three separate calls in a sequence after being exposed to playbacks. While we assumed initially that a feeding context may act as a reinforcer to learn calls, our data showed the opposite effect, with learning more apparent in the animals that received playbacks not specifically linked with their nursing activities. Nursing had already begun when we started playbacks for nursing pups, and it is possible that seals only focus on their mothers at that time. Nursing may have influenced the total number of calls recorded from pups but this was only a minor effect since most calls came from times before and after nursing. The fact that seals acquired the playback calls rather than converging on the call of another pup in the area is likely a result of the consistency and lack of variation in our playbacks in contrast to the greater intra-individual variation in natural pup calls. However, our results suggest that in the absence of playbacks pups adjust their calls to those of other pups.

It is unclear why vocal learning might be used by grey seals to produce conformity in pup calls at a haul-out site. Pup calls are not part of the adult repertoire [16,20] and pups stay relatively stationary on land throughout their infancy so that group recognition is not a likely explanation. Furthermore, grey seals at our study site do not use vocal parameters for mother–pup recognition [16]. However, learning may also affect the development of the adult repertoire so that learned variation in pup calls may just be a by-product or early manifestation of learning abilities relevant for the development of adult repertoires. Adult female grey seals tend to associate across seasons [21] and there is evidence for mate fidelity [22]. Learned call conformity may help to maintain such relationships and to facilitate recognition. Learned conformity at breeding sites can also lead to cultural variation in calls across locations. Such vocal variation between geographically distant areas has been described for a variety of pinniped species [23,24]. Grey seals show pupping site fidelity [25], but it is unclear whether cultural differences in vocalizations play a role in this pattern.

Our study is the first experimental test of mammalian vocal learning in the field to our knowledge. By creating or changing the acoustic environment around pups, we could alter their vocal behaviour while leaving them in their natural environment. We hope that this method can be used in other mammalian species to test vocal learning. However, additional social stimuli might be necessary to induce learning in other mammalian orders. It appears that pinnipeds use vocal learning in a variety of contexts. Male elephant seals, the only other pinnipeds in which vocal learning has been studied in the wild, have been found to use the same call parameters as successful males [26] and vocal learning may be important in song development [23,24]. Thus, intra- and intersexual selection as well as social recognition and the maintenance of social bonds could have contributed to its evolution in pinnipeds.

## Data accessibility

The research data underpinning this publication can be accessed at https://doi.org/10.17630/080c0f26-0a9e-4483-ab12-ab8985191371 [27].
